# Enhanced Objective Detection of Retinal Nerve Fiber Bundle Defects in Glaucoma With a Novel Method for En Face OCT Slab Image Construction and Analysis

**DOI:** 10.1167/tvst.10.12.1

**Published:** 2021-10-04

**Authors:** Riccardo Cheloni, Simon D. Dewsbery, Jonathan Denniss

**Affiliations:** 1 School of Optometry and Vision Science, University of Bradford, Bradford, UK; 2 Department of Ophthalmology, Leeds Teaching Hospitals NHS Trust, Leeds, UK

**Keywords:** glaucoma, optical coherence tomography, en face, retinal nerve fiber layer, reflectance

## Abstract

**Purpose:**

To introduce and evaluate the performance in detecting glaucomatous abnormalities of a novel method for extracting en face slab images (SMAS), which considers varying individual anatomy and configuration of retinal nerve fiber bundles.

**Methods:**

Dense central retinal spectral domain optical coherence tomography scans were acquired in 16 participants with glaucoma and 19 age-similar controls. Slab images were generated by averaging reflectivity over different depths below the inner limiting membrane according to several methods. SMAS considered multiple 16 µm thick slabs from 8 to 116 µm below the inner limiting membrane, whereas 5 alternative methods considered single summary slabs of various thicknesses and depths. Superpixels in eyes with glaucoma were considered abnormal if below the first percentile of distributions fitted to control data for each method. The ability to detect glaucoma defects was measured by the proportion of abnormal superpixels. Proportion of superpixels below the fitted first percentile in controls was used as a surrogate false-positive rate. The effects of slab methods on performance measures were evaluated with linear mixed models.

**Results:**

The ability to detect glaucoma defects varied between slab methods, χ^2^_(5)_ = 120.9, *P* < 0.0001, with SMAS showing proportion of abnormal superpixels 0.05 to 0.09 greater than alternatives (all *P* < 0.0001). No slab method found abnormal superpixels in controls.

**Conclusions:**

SMAS outperformed alternatives in detecting abnormalities in eyes with glaucoma. SMAS evaluates all depths with potential retinal nerve fiber bundle presence by combining multiple slabs, resulting in greater detection of reflectance abnormalities with no increase in surrogate false positives.

**Translational Relevance:**

SMAS may be used to objectively detect glaucoma defects in en face optical coherence tomography images.

## Introduction

Optical coherence tomography (OCT) is increasingly used to assess structural changes of the retina owing to glaucoma.^1^^–^^3^ Such changes are conventionally evaluated in cross-sectional scans assessing the thickness of either the retinal nerve fiber layer (RNFL) or the ganglion cell and inner plexiform layers.^2^^,^^4^ En face OCT imaging is a relatively new approach that uses transverse retinal images to assess reflectance properties of retinal nerve fiber bundles (RNFBs).^5^ Compared with assessing RNFL reflectance in fundus photographs,^6^^,^^7^ en face OCT has advantages in better visualization of narrow defects and preserved bundles, the ability to examine below the superficial RNFL, and being less affected by lens opacities and light fundus pigmentation.^8^^–^^10^ En face OCT analysis of reflectivity has also demonstrated potential for early glaucoma detection^11^^–^^13^ and is a potential means to facilitate custom perimetry strategies that target specific regions of interest.^14^^–^^18^ Although direct observation of RNFBs may be beneficial, optimal methods to construct en face slab images are yet to be determined, and automated, objective methods to detect glaucoma defects in this domain are also lacking.^13^^,^^19^

En face images are usually generated from dense volumetric scans of the area of interest followed by the projection of pixel intensities from a certain range of depths within each A-scan into a transverse slab image.^20^ Here, healthy RNFBs appear hyper-reflective because of the ordered structure of their axonal cytoskeletons,^21^ and this property may be lost early in glaucoma, decreasing reflectivity.^22^ Thinning of the RNFL leads to inclusion of deeper, hyporeflective retinal layers in the slab image, also decreasing reflectivity.^5^^,^^23^ Damaged bundles, therefore, appear in en face images as loss of reflectivity following typical patterns such as arcuate and wedge-shaped defects.^24^

There are several possible approaches to en face image extraction, including variations in the region of retina imaged and the composition of slabs as defined by the depths below the inner limiting membrane (ILM) over which A-scan pixels are averaged. Furthermore, different arithmetic methods to convert three-dimensional data into transverse images and different approaches to account for individual anatomic variability may be considered. We previously showed that the configuration of RNFBs in healthy eyes varies with retinal location and individual anatomy.^19^ Accordingly, the final appearance and diagnostic usefulness of en face slab images is likely to be affected by the methods used for slab construction. These effects have been investigated minimally and therefore choices are currently made based on limited information.

Previous work in this area has often averaged the first 50 µm below the ILM in a single slab image.^5^^,^^23^^,^^25^^,^^26^ Consistently with RNFL thickness,^27^^,^^28^ RNFBs are present at depths of more than 50 µm proximal to the optic nerve head (ONH),^19^ and limiting en face analysis to this depth might, therefore, miss early glaucoma defects in some regions.^24^ Further, although several authors have recognized a need to adjust slab characteristics to individual anatomy and the varying morphology across the retina,^5^^,^^23^^–^^25^ these adjustments have not been fully considered.

In this study, we introduce summary of multiple anatomically adjusted slabs (SMAS), a novel method for the construction and analysis of slab images. SMAS aims to address current limitations of en face imaging, including (i) adapting to individual anatomy, (ii) considering all depths and regions that contain visible RNFBs in healthy eyes, and (iii) adjusting for different layer morphology across the retina. We also evaluate the ability of several alternative slab construction methods to objectively detect glaucoma defects as compared with SMAS.

## Methods

### Participants

Twenty-two participants with open angle glaucoma and 19 age-similar healthy controls were recruited for this study. All participants underwent ophthalmic examination including subjective refraction, slit lamp assessment, Goldmann applanation tonometry, retinal OCT (Spectralis, Heidelberg Engineering, Heidelberg, Germany) and standard automated perimetry (24-2 SITA-Standard, Humphrey Field Analyzer III, Carl Zeiss Meditec Inc., Jena, Germany). Participants with glaucoma were only included if older than 40 years and presenting a clinical diagnosis of open angle glaucoma. In addition, inclusion required evidence of structural damage defined as at least one ONH sector with *P* < 1% from the Spectralis circumpapillary RNFL thickness analysis. No visual field inclusion criteria were applied to the glaucoma group to include the earliest cases. Participants with refractive error magnitude greater than 6.00 DS or 3.00 DC, evidence of lens opacification,^29^ or other eye conditions except glaucoma were excluded. Healthy participants were included if they had no eye disease or history of eye disease and normal visual field as defined by a normal Mean Deviation (*P* > 5%), glaucoma hemifield test within normal limits and absence of three contiguous non-edge points with *P* < 5% on the pattern deviation plot. One eye per participant was included. If both eyes were eligible, the tested eye was selected at random in healthy controls, whereas the one with milder defect (less negative Mean Deviation) was included in participants with glaucoma.

All participants provided written informed consent to participate and were free to withdraw at any time. The study adhered to the tenets of the Declaration of Helsinki and achieved ethical approval from the National Health Service's Research Ethics Service.

### OCT Imaging and Image Processing

Seven high-speed dense OCT scans were taken of the central ±25° of the retina (30 µm separation between B-scans) of each participant. The OCT procedure used has been described in detail previously.^13^^,^^19^ All images were acquired with signal to noise ratio of greater than 20 dB as recommended by the manufacturer.

Single-pixel deep slabs (*n* = 50) of the instrument's maximum digital axial resolution (3.87 µm), ranging from the ILM to 193.5 µm below it, were extracted from individual B-scans. Slab images were converted to depth-resolved attenuation coefficients,^30^ which represent an intrinsic optical property of the retinal tissue^31^ and have been advocated to minimize the impact of artefacts in en face images.^24^ Attenuation coefficient data were imported into MATLAB (Version 9.6.0, The MathWorks Inc., Natick, MA) for montaging and image processing. Before montaging, gamma correction was used to smooth intensity differences between OCT images from different retinal locations by matching the luminance of overlapping regions of neighboring images at each depth using the central macular image as the reference.^32^ For this gamma correction, we used the ratio between the median intensities of the individual slab and the macular image in corresponding overlapping regions as the gamma coefficient. We then montaged the images using custom software, again using the macular scan as a reference image. The highest pixel value was selected from overlapping regions.

Montaged images were processed as described in detail previously.^19^ In brief, the intensity of an area with no RNFBs within the raphe region was extracted 35µm below the ILM, and set as background with a threshold transformation. Then, pixel intensity was normalized by dividing by the mean of the 99th percentiles from each depth. Values were clipped to 1, resulting in images with pixel intensities in the range of 0 to 1.

### Adjusting for Individual Anatomy

**Figure 1.**
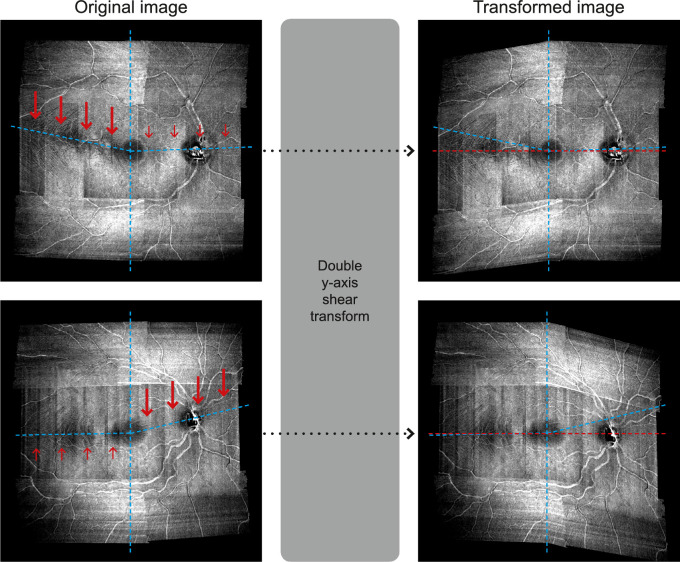
Example of the double vertical shear transformation applied to the en face images of two healthy controls. For the image shown in the *top panels*, the major transformation (*thick red arrows*) was applied to the temporal retina whereas a more minor transformation (*small red arrows*) was applied to the nasal retina. The opposite applies for the image shown in the bottom panels. Irrespectively of the original anatomy (*blue dashed lines*), transformed images (*right column*) align the raphe, fovea, and ONH along a horizontal line (*red dashed lines*).

We aimed to minimize the impact of individual anatomy by adjusting en face images to the fovea–disc and fovea–raphe angles. We used geometric image transformations to align the ONH, fovea, and raphe along a common horizontal axis (Fig. 1). Left eyes were flipped to right eye format and the slab image offering best visibility of the foveal pit was used to manually extract the coordinates of the fovea and the center of the ONH. The fovea–disc angle was defined as a straight line between these two points. The orientation of the raphe is known to change with individual anatomy and can be measured in both healthy eyes and eyes with glaucoma.^33^ Accordingly, the fovea–raphe angle was extracted following an existing method.^34^ Using the single slab image with best visibility of RNFBs in the raphe area (median, 15.5 µm below the ILM; range, 11.6–27.1 µm), the fovea–raphe angle was measured by tracing lines connecting the fovea to five manually selected points in the raphe gap region. We took the average of the five angles identified as the fovea–raphe angle.^34^ We then aligned the raphe, fovea and ONH along a horizontal line by applying vertical shear transformations separately to the image regions either side of the fovea (Fig. 1). Shear transformations enable the shift of a single dimension of the image (vertical in this case) by a given angular value, leaving the other (horizontal) dimension unmodified. This approach enabled evaluation of reflectivity over a square array of superpixels (discussed elsewhere in this article), with landmark retinal locations laying on a common horizontal axis (Fig. 1).

### Extraction of Reflectance Abnormalities With SMAS

With the objective of considering all depths with present RNFBs and the differing layer morphology across the retina, we averaged together groups of four single-pixel deep slabs starting from 7.8 µm up to 193.5 µm below the ILM. The first two depths (i.e., <7.8 µm below the ILM) were excluded from slab construction because they do not contain visible RNFBs in healthy eyes,^19^ but are likely to contain vitreous interface and glial artefacts.^24^^,^^35^ The averaging of groups of four slabs together aimed to combine sufficient single pixel slabs to minimize image noise while also minimizing the mixture of retinal layers (i.e., RNFL with deeper layers such as ganglion cell and inner plexiform layers).^19^ RNFBs are visually present at the narrowest range of depths in the temporal retina, and the averaging adopted by SMAS should allow inclusion of all bundles from these regions in the first slab (from 7.8 µm to 23.2 µm below the ILM). This goal should also be achieved in eyes with particularly thin RNFL.^19^ This process yielded 12 slabs (each approximately 15.5 µm thick) from 7.8 µm to 193.5 µm below the ILM (Supplementary Table S1).

**Figure 2.**
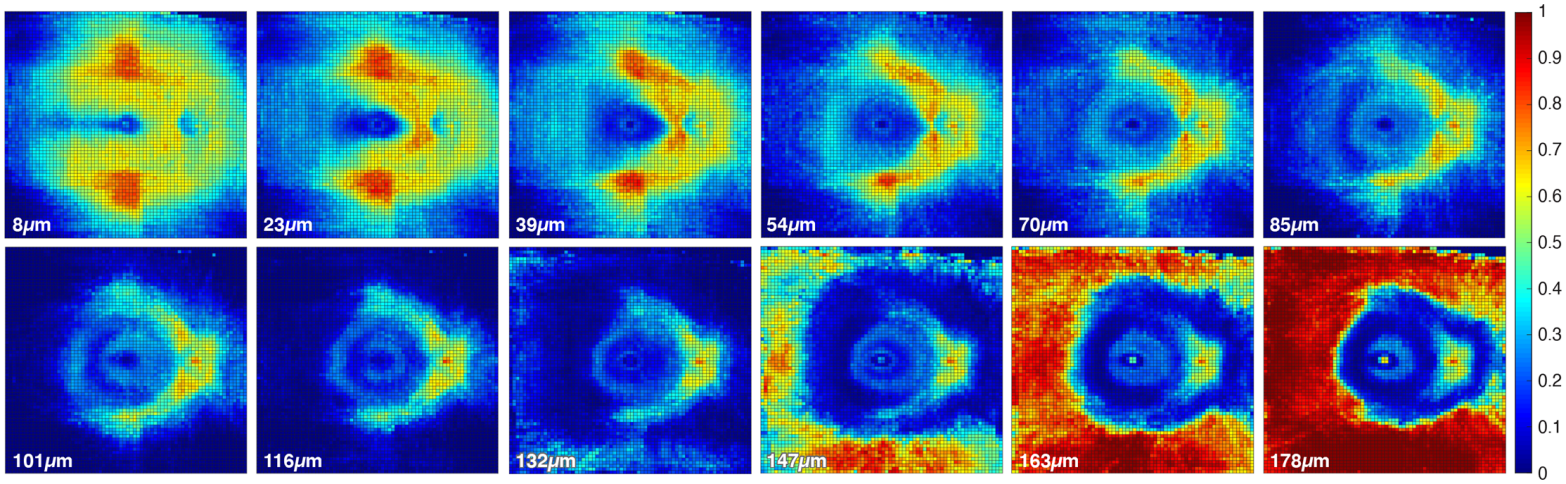
Heat maps of median normative data for the 12 slabs generated in the SMAS method. It is possible to identify the main retinal structures such as the temporal raphe, fovea, and ONH. At greater depths the hyper-reflectivity of the retinal pigment epithelium becomes visible. Depths (in micrometers) shown correspond with the anterior depth at which each of the 15.5-µm-thick slabs commenced. The heat maps also show hyper-reflective artefacts in the superior- and inferior regions (first 3–4 slabs), likely owing to the varying beam light incident angles from wide-field OCT imaging (see Discussion).^37^

**Figure 3.**
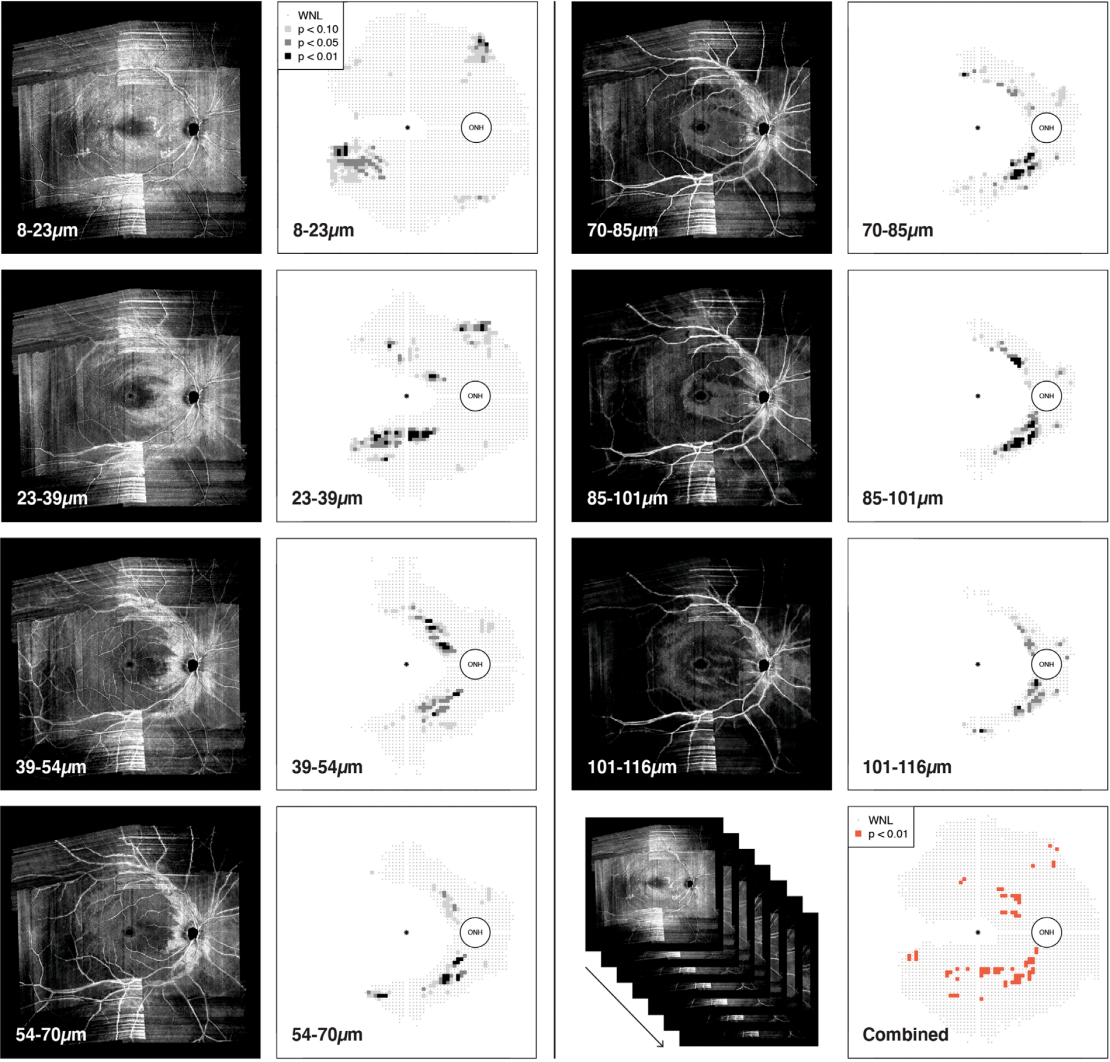
Example of the multiple slab images generated with the SMAS method for one participant with glaucoma, as well as the combined deviation map reporting all reflectance abnormalities detected in any slab. At each specific slab depth, whose starting and ending depths are reported in micrometers, the left-most image shows the actual slab image and the right-most image shows the corresponding deviation map. In deviation maps grey points indicate superpixels found within normal limits (WNL), and superpixels below the tenth, fifth, and first percentiles are reported as squares color-coded according to the level of significance. The bottom-right panel shows the combined deviation map with abnormal superpixels (<1%) identified at any depth. In deviation maps, each data point corresponds with 1 superpixel.

An analysis of reflectivity was performed on a superpixel grid centered on the fovea, with each superpixel composed of a number of individual pixels in a *n* × *n* pixel configuration. The intensity of each superpixel was the mean of its constituent pixels. Additional mitigation of anatomic variability was achieved by controlling for the varying distance between the fovea and ONH by adjusting the size of superpixels in individual images such that a fixed 20 superpixels separated the fovea and ONH. This number was chosen to target a superpixel dimension of 20 × 20 pixels, previously suggested to represents a suitable compromise between between-subject variability and sufficient resolution to detect wedge shaped defects.^24^ Superpixels in individual images contained median 20 × 20 pixels (range, 17 × 17 to 23 × 23) and this system of coordinates maximized consistency between retinal locations among different eyes. See Figures 2 and 3 for examples of the superpixel grid.

The distributions of superpixel intensities at all depths in control eyes were explored visually at different retinal locations and Shapiro–Wilk tests for normality were performed. The majority of distributions were either multimodal or significantly skewed. To account for the observed distributions and the modest sample size, summary statistics and limits of normality were derived from kernel density-estimated frequency distributions rather than the empirical data.^36^ We extracted the estimated median (Fig. 2), tenth, fifth, and first percentiles at all depths of all superpixels.

As reported previously,^19^ and as shown in Figure 2, the presence of RNFBs throughout the retina varies with retinal location and depth below the ILM. An evaluation of the reflectance in locations where RNFBs are not expected to be visible even in healthy retinae would have no diagnostic value; therefore, these areas were censored from analysis. Accordingly, analysis was restricted to regions of interest in the first 7 slabs (up to 116 µm below the ILM). Regions of interest were manually identified as those containing visible RNFBs in the control eyes. For each depth individually, a value 2.5 standard deviations below the grand mean intensity within regions of interest across all control images was set as a threshold, and regions with a lower mean intensity in control eyes were excluded from analysis in all images. This threshold (2.5 standard deviations below the mean) was chosen as the best compromise between maximizing the retinal area evaluated and the adequate exclusion of regions with no visible RNFBs among several cut-offs trialed (Supplementary Fig. S1).

Finally, slab images were extracted in all participants with glaucoma according to the SMAS approach. Superpixel values within the previously defined regions of interest at each depth were compared with corresponding normal limits from controls. Reflectance abnormalities were identified in single depth deviation maps because intensities below the estimated tenth, fifth, and first percentiles of control data (Fig. 3). The seven deviation maps from each depth were then combined into a summary deviation map reporting abnormal superpixels (<1%) identified at any depth (Fig. 3, bottom right).

### Extraction of Reflectance Abnormalities With Alternative En Face Slab Methods

**Figure 4.**
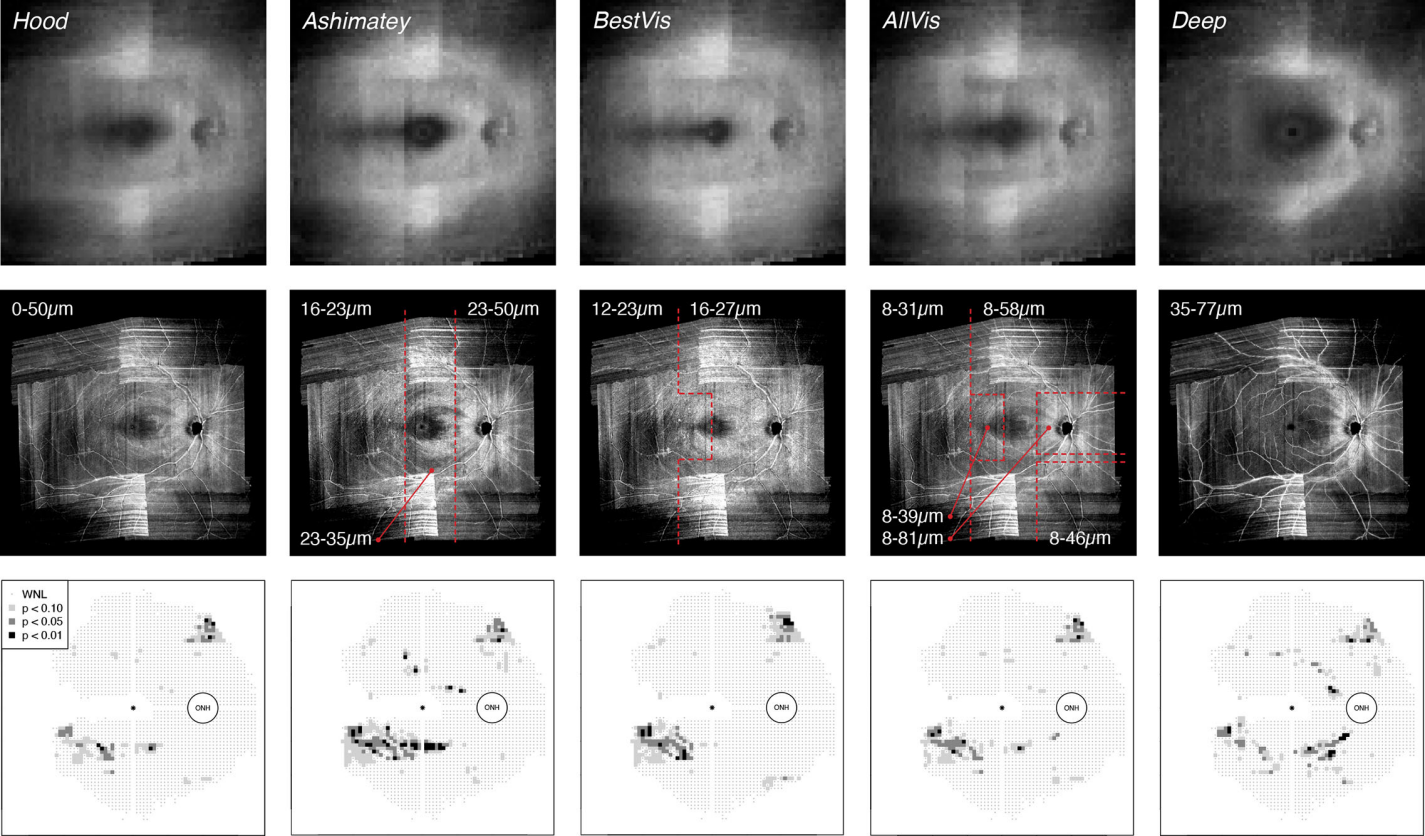
Alternative methods of slab construction explored in this study in addition to SMAS. (*Top row*) Method-specific normative en face slabs derived from control eyes. (*Middle row*) Individual slab images for the same participant with glaucoma as shown in Figure 3. Red dashed lines demarcate different regions of the slab characterized by different depths considered (see text), as labelled in µm. (*Bottom row*) Corresponding deviation maps for different slab methods. Format of deviation maps as per Figure 3.

En face images for all participants were also generated using several other slab methods based primarily on previous studies.^5^^,^^19^^,^^24^ For each method, transformed single pixel slabs of individual eyes were averaged together over a specific range of depths. Normative data from controls were then extracted as described earlier and reflectance abnormalities were evaluated in eyes with glaucoma using the method described elsewhere in this article. Adjustments for individual anatomy made for SMAS were applied identically for other slab methods, as was the positioning and spacing of the superpixel grid. Therefore, different slab methods differed only in the retinal depths averaged (Fig. 4). All slab methods were evaluated over the same region of the retina tested by SMAS.

#### Hood Slab

This method was similar to that of Hood et al.,^5^ in which the pixel intensity was averaged over a 52-µm deep slab starting from the ILM. We averaged the first 13 single-pixel slabs together, encompassing depths from the ILM to 50.3 µm below the ILM as the closest possible match to the method of Hood et al.^5^

#### Ashimatey Slab

Ashimatey et al.^24^ used a slab with decreasing thickness from the ONH to the temporal retina. They averaged pixel intensity from 24 to 52 µm below the ILM in the optic disc region, from 24 to 36 µm in the central retina and from 16 to 24 µm in the temporal macula and raphe region. To reproduce a similar slab configuration, we considered three vertically separated regions with different thickness (Fig. 4): the 7th through the 13th pixels in the ONH region (approximately 23.3 to 50.3 µm), the 7th to the 9th pixels in the macular area (approximately 23.3 to 34.8 µm), and the 5th to the 6th pixels in the raphe area (approximately 15.6 to 23.2 µm).

#### Best Visibility Slab (BestVis)

This slab was centered on the depth of best RNFB visibility across the retina in healthy eyes, which we found previously at an average of 20.3 ± 1.9 µm below the ILM, with slight differences between the temporal and nasal retina.^19^ Accordingly, the single pixel slab at the depth of best RNFB visibility was averaged together with the one above and the one below in the BestVis slab (Fig. 4). The fourth through the sixth pixels were included in the raphe and temporal macula (approximately 11.7 to 23.2 µm; best = 18 µm), whereas the fifth through the seventh pixels were averaged in the rest of the retina (15.6 to 27.1 µm; best = 22 µm).

#### All Visible RNFBs Slab (AllVis)

As per the best visibility slab, this approach considered our previous work^19^ and averaged all depths expected to contain visible RNFBs in healthy eyes. Differences in RNFB visibility across the healthy retina were accounted for by averaging varying depths in different regions of the retina (Fig. 4). Hence, the pixel depths included were the 3rd to 8th in the raphe (approximately 7.8 to 31 µm); 3rd to 10th in the temporal macula (approximately 7.8 to 38.7 µm); 3rd through 12th in the inferior nasal quadrant (approximately 7.8 to 46.4 µm); 3rd through 15th in the central and superior-nasal retina (approximately 7.8 to 58.1 µm); and 3rd through 21st in the ONH region (approximately 7.8 to 81.3 µm).

#### Deep Slab

The deep slab included greater depths below the ILM than considered by most of the methods discussed elsewhere in this article and was included as a control. The deep slab averaged intensity starting from depths close to the posterior limit of the Hood slab and the Ashimatey slab up until the greatest depths at which arcuate regions and the nasal retina around the ONH still present RNFBs in healthy eyes.^19^ The slab averaged the 10th to 20th pixels through the whole retina, corresponding with 34.9 to 77.4 µm below the ILM.

### Analysis

All extracted slabs and corresponding deviation maps were examined by two authors (RC, JD) for the impact of artefacts. Either whole images or specific regions from participants with substantial effect of artefacts were excluded from further analysis. Artefacts of en face images could arise from low-quality B-scans, floaters, and glial cell alterations.^35^ A joint discussion of single cases was performed until a consensus on data exclusion was reached.

The performance of different slab methods was compared by the proportion of abnormal superpixels identified in each participant with glaucoma. This metric was computed as the number of superpixels below the first percentile of the corresponding normative data divided by the number of tested superpixels. For SMAS, the combined deviation map was considered. The differences in the proportion of abnormal superpixels between slab methods were explored with linear mixed models and χ^2^ likelihood ratio tests*.*^38^ The slab method was considered as a fixed effect, whereas individual participants were modelled as random effects to account for the repeated-measures design. Statistical significance was considered at *P* < 0.5 and the model had the following form:

(1) $$y∼1+Slab\;Method+\left(1|Eye\right)+ɛ\left[6pt\right]$$

where *y* signifies the outcome of interest (e.g., the proportion of abnormal superpixels), and 1 and ε signify intercept and random error, respectively. Pairwise differences were tested with post hoc *t* tests, adjusting for multiple comparisons with the Tukey method.

To further characterize individual slab methods, the median distance of abnormal superpixels from the ONH was extracted from all participants with glaucoma for all different approaches. Because specific methods might include different retinal sections across the area examined, the resulting slab composition and therefore the ability to detect abnormalities could also change with retinal location.

False-positive rates of different slab methods could not be evaluated directly owing to the lack of an independent reference standard. As a surrogate measure, we explored the rate of abnormal superpixels in control eyes at the 1% level of significance (derived from kernel density estimation as described earlier). Linear mixed models of the form above were used to evaluate differences of distance from the ONH and surrogate false-positive rate among different slab methods. Last, we tested whether differences in performance between SMAS and alternatives were related to the severity of reflectance defects. As such, we computed coefficients of determination (R^2^) between the mean and standardized difference in proportions of abnormal superpixels between each slab method and SMAS.

As estimated with the *simr R* package,^39^ this study had 91% power (95% confidence interval [CI], 89.2–92.8) to detect a 0.02 difference in the proportion of abnormal superpixels at an alpha of 0.05.

## Results

Images from six eyes with glaucoma (median age, 69 years; range, 67–78 years; median Mean Deviation, –6.0 dB; range, –1.6 to –12.8 dB) contained significant artefacts and were excluded from the main analysis. Further, part of the en face images of three participants with glaucoma were censored for similar reasons. For one participant, the whole upper hemifield was excluded, whereas a horizontal band in the upper retina and the inferior temporal retina were censored in the remaining two cases. Overall, 19 controls (median age, 68 years; range, 56–75 years) and 16 participants with glaucoma (median age, 70 years; range, 61–77 years) were included. All participants in the glaucoma group but one had a visual field defect according to the definition used for the control group's exclusion criteria. The remaining participant with glaucoma had three contiguous defective points, but one was an edge location. Table reports detailed participant demographics.

**Table.** Demographics of Included Participants

	Control Group	Glaucoma Group
n	19	16
Age (years)	68 (6)	70 (8.25)
Caucasian/other ethnicity	18/1	16/0
Male/female	8/11	8/8
Standard automated perimetry Mean Deviation (dB)	0.8 (1.4)	−3.3 (2.2)
Average cpRNFL thickness (µm)	98 (11.5)	68 (14)
Axial length (mm)	23.26 (0.50)	24.12 (0.96)

cpRNFL, circumpapillary retinal nerve fiber layer.

Continuous data are reported as median and (interquartile range).

**Figure 5.**
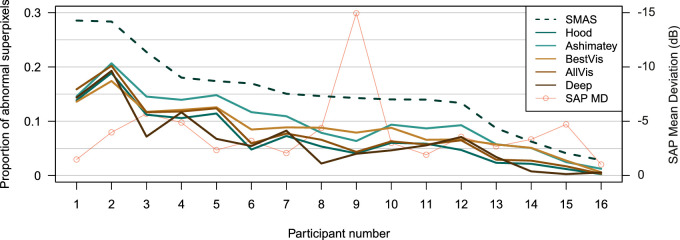
Proportion of abnormal superpixels identified by different slab methods in all participants with glaucoma. Visual field mean deviation is also shown. Images from participants 1, 6, and 8 were partially censored owing to artefacts.

Figure 5 shows the proportion of abnormal superpixels identified by different slab methods for all participants with glaucoma. SMAS found a greater proportion of abnormal superpixels than all alternative slab methods in all participants with glaucoma.

**Figure 6.**
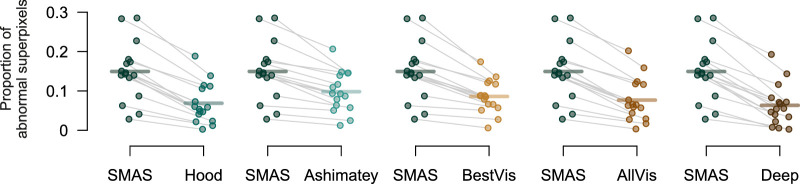
Differences of proportion of abnormal superpixels between each single slab method and SMAS. Different methods are color coded as per Figure 5. Grey lines link data from individual images, horizontal lines indicate means. All differences *P* < 0.0001.

There were significant differences between slab methods in the proportion of abnormal superpixels identified in the eyes with glaucoma, χ^2^_(5)_ = 120.9; *P* < 0.0001. Pairwise differences between SMAS and each other slab method are shown in Figure 6. All other methods identified smaller proportions of abnormal superpixels compared with SMAS (all *P* < 0.0001). The smallest difference in proportion of abnormal superpixels to SMAS was found for the Ashimatey slab (–0.051; 95% CI, –0.063 to –0.039; *P* < 0.0001), whereas the deep slab showed the largest difference (–0.086; 95% CI, –0.098 to –0.074; *P* < 0.0001). Among alternative methods, the Ashimatey slab performed significantly better than the Hood, AllVis, and deep slabs by 0.03 (95% CI, 0.018–0.042), 0.022 (95% CI, 0.01–0.034), and 0.035 (95% CI, 0.023–0.047), respectively (all *P* < 0.05), whereas the BestVis slab outperformed the deep slab by 0.023 (95% CI, 0.011–0.035; *P* = 0.005).

**Figure 7.**
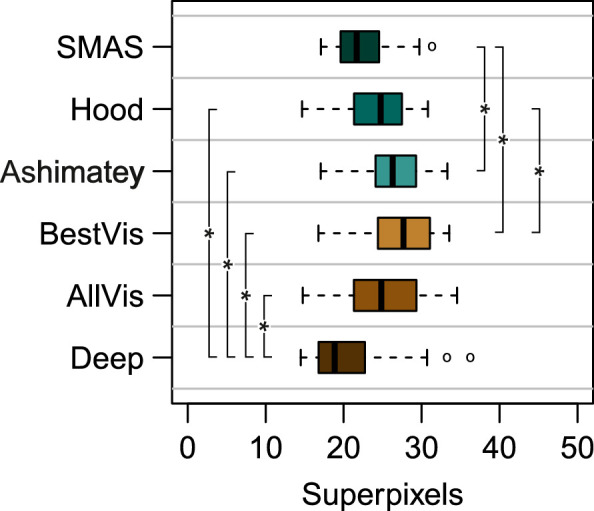
Boxplot showing the median distance from the center of the ONH of abnormal superpixels in eyes with glaucoma by slab method. Data are color coded according to different slab methods as per previous figures. Slab methods showing statistically significant differences (*P* < 0.05) of defect distance from the ONH are flagged with (*).

The median distance of abnormal superpixels from the ONH differed significantly between different slab methods, χ^2^_(5)_ = 50.0; *P* < 0.0001. As shown in Figure 7, the median distances from the ONH of abnormal superpixels using the Ashimatey and Best Visibility slabs were greater than those for SMAS and the deep slab. The median distance of abnormal superpixels from the ONH for the Ashimatey and Best visibility slabs were significantly greater than for SMAS (differences, 3.5 superpixels [*P* = 0.003] and 4.6 superpixels [*P* < 0.0001], respectively). The median distance of abnormal superpixels found with the deep slab was closer to the ONH than all other slab methods (all *P* < 0.05) apart from SMAS, for which distances were smaller but statistically similar (deep - SMAS, –1.6; *P* = 0.47).

We computed the rate of abnormal superpixels in controls as a surrogate measure of the false-positive rate. None of the slab methods showed superpixels with intensity below the first percentile of control eyes, which was the cut-off used to define defects in the glaucoma group.

**Figure 8.**
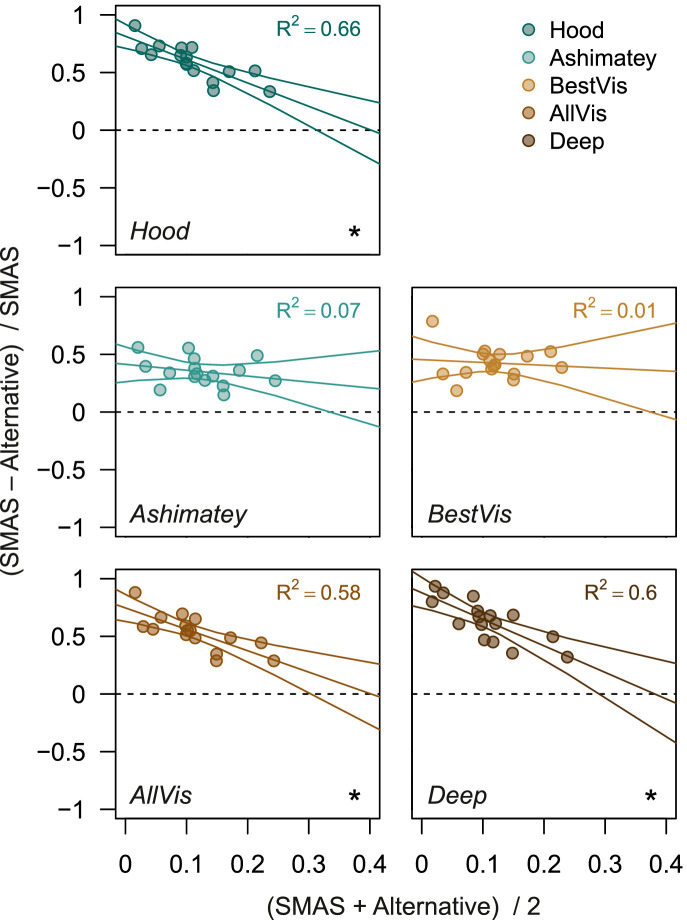
Bland-Altman–like scatter plots assessing the relationship between the mean and standardized difference of proportion of abnormal superpixels between SMAS and alternative slab methods. Standardized difference was calculated as the difference between proportion of abnormal superpixels of SMAS and the alternative slab method, divided by the proportion of abnormal superpixels of SMAS. The best linear fit to the data (including 95% CI) and corresponding R^2^ are also reported. Cases where the linear regression line presented a slope significantly different from 0 (*P* < 0.05) are flagged with (*). Different slab methods are color-coded as per previous figures.

Scatterplots showing the relationship between the mean and standardized differences in proportion of abnormal superpixels between SMAS and each of the other slab methods are shown in Figure 8. The Hood, AllVis, and deep slabs showed a negative relationship between differences in detection of reflectance abnormalities and average reflectance loss (slopes *P* < 0.01; R^2^ 0.58–0.66). This finding suggests that the benefit of SMAS over these alternatives is greater for earlier defects. The same was not true for the Ashimatey and BestVis slabs*,* whose performance compared with SMAS was relatively consistent across the range of reflectance loss in this sample.

## Discussion

There are many possible ways to construct en face slabs from OCT images, and there is currently limited evidence on optimal slab construction methods for detection of glaucomatous defects. In addition to slab construction, there is also a lack of strategies for automated, objective definition of defects, which should ideally account for anatomic variation between individuals. Such strategies may facilitate the consistent evaluation of reflectance loss, as well as the use of en face findings for seeding other investigations, such as custom perimetry.^14^^,^^18^^,^^40^^,^^41^ In this study, we introduced SMAS, a novel approach for the construction of en face slab images and the automated, objective detection of glaucomatous defects in the en face images. SMAS improves on existing methods in several ways, such as (i) examination of all depths that contain visible RNFBs in healthy eyes; (ii) greater consideration of the varying configuration of RNFBs across the retina; (iii) greater consideration of interindividual anatomic variability; and (iv) consideration of a wider area of the retina to include all regions containing visible RNFBs in healthy eyes, except the temporal raphe.

Compared with other methods, SMAS detected a greater proportion of abnormal superpixels in eyes with glaucoma (Fig. 6). Although SMAS was the only method to combine information from multiple separate depth slabs, our analysis of surrogate false positive rate yielded no abnormal superpixels in control eyes at the cutoff used to identify defects in eyes with glaucoma, the same as the other slab methods. This finding suggests that SMAS detects more defects in eyes with glaucoma without increasing the number of false positives in healthy eyes.

The increased detection of abnormal superpixels in eyes with glaucoma with SMAS is attributable to the consideration of multiple slabs through the range of depths containing visible RNFBs in healthy eyes, because the greater retinal area and novel treatment of anatomic variability was applied to all slab methods in this study. The adjustments for anatomic variability were applied to all slab methods, even though individual anatomy was not adjusted for in the same way in the original applications of these methods.^5^^,^^23^^,^^24^ This adjustment for individual anatomy could be expected to decrease the variability of measurements and increase the retinal area available for assessment.^24^

SMAS aimed to evaluate changes of reflectivity at all retinal depths and regions that contain visible RNFBs in healthy eyes. The assessment of the full range of depths accounts for the increased proportion of defects compared with the Ashimatey and BestVis slabs, which both assess relatively thin slabs restricted to the first 20 to 50 µm below the ILM. Accordingly, these two approaches might be as effective as SMAS at detecting reflectance changes in retinal regions with physiologically thin RNFL where they capture the full thickness. Figures 3 and 4 exemplify this. In Figure 3, an inferior arcuate defect is visible starting from 39 µm below the ILM. The BestVis and Ashimatey slabs completely or partially failed to detect this defect, although the defect's origin at the ONH was instead well-depicted in the deep slab (Fig. 4). The median distances of abnormal superpixels from the ONH conform with this interpretation; the BestVis and Ashimatey slabs on average detected abnormal superpixels further away from the ONH, where the RNFL is thinner and RNFBs are present at a smaller range of depths below the ILM.^19^^,^^27^ Conversely, the deep slab examined depths that only contain RNFBs in the nasal retina and unsurprisingly found defects significantly closer to the ONH by 5.1 and 6.2 superpixels, respectively (both *P* < 0.0001).

In SMAS, the depth-averaging of only 4 pixels (approximately 16 µm) per individual slab minimizes contamination of slab images by deeper retinal layers that do not contain RNFBs, even in the temporal region where the RNFL is thinnest.^19^ The greater depth-averaging of other methods (e.g., the Hood and AllVis slabs) may be more prone to between-individual variability leading to more variable normative reflectivity data, ultimately impacting the ability to detect glaucomatous changes. This hypothesis is supported by the greater identification of defects by SMAS compared with the AllVis slab, which assessed approximately the same retinal depths but with depth-averaging across the whole depth assessed.

To our knowledge, this study is the first attempt to quantify the ability of different en face slab construction methods to detect changes of reflectivity owing to glaucoma. In the few previous studies, authors have usually based their slab construction method on pilot testing only.^5^^,^^23^^–^^25^ Direct comparison of our results with previous work is complicated by differing study aims, methods, OCT devices, retinal area examined, and populations. Further, most studies performed subjective evaluation of reflectance abnormalities^5^^,^^23^^,^^25^ and, to our knowledge, the only previous analysis including an objective extraction of glaucoma en face defects is the work of Ashimatey et al.^24^ Hood et al considered a smaller region centered on the ONH,^5^ whereas other investigators mainly focused on the macula.^23^^,^^25^ Different target regions would result in different configurations of the RNFBs in the area tested, justifying the selection of different parameters. Notwithstanding the difficulties in direct comparison, our results are broadly in line with those of previous studies. In our own previous work, we showed that RNFB configuration varies across the retina, suggesting that slab parameters should be adjusted to detect defects consistently across the retina.^19^ This finding was confirmed in this study, showing that slab methods do affect the capability to identify defects. Ashimatey et al.^24^ noted that the inability of their slab method to identify all reflectance losses and the requirement to extend the analysis further below 52 µm to retrieve all defects. Further work from the same lab is the only previous attempt to analyze the effect of different slab construction parameters on the detection of glaucoma defects.^42^ That study considered the average reflectance of small circular regions (30 pixels diameter) placed around the ONH with different ranges of depths combined together in several slabs. The greatest ability to detect glaucoma was achieved by averaging reflectivity from 36 to 60 µm below the ILM, as compared with slabs of 0 to 52 µm, 24 to 52 µm, and 24 to 36 µm.^42^ These results confirm the importance of considering greater depths with present RNFBs to retrieve glaucoma defects. However, the inclusion of greater depths should not be achieved by averaging across large depths of retina, but rather with alternative approaches able to preserve consistency of slab composition.

This study has limitations. Although we included processing strategies to adjust for uneven illumination of scans from different retinal locations and computed attenuation coefficients to minimize the impact of artefacts, the final images were still affected by these issues. Indeed, some eyes had to be excluded owing to a substantial impact of artefacts, from either activated glial artefacts, uneven illumination and/or low quality B-scans. More sophisticated image processing and/or improved image capture may further decrease the impact of such artefacts in the future. The consideration of the varying incident light beam angle at the OCT image acquisition stage would also likely improve en face OCT analyses, especially when imaging the wider retina.^37^^,^^43^^,^^44^ Furthermore, the small sample size did not allow for an exploration of the impact of different parameters on the observed reflectance, such as age, eye laterality or ethnicity.^28^^,^^31^^,^^45^ Larger studies could allow the development of normative data adjusted for covariates with clinically significant impact on reflectivity, ultimately leading to further refinement of the slab extraction method.

An additional constraint on our study design is the lack of an appropriate reference standard for identifying whether superpixels flagged as defective are flagged correctly or not. Accordingly, the performance of each slab method could not be evaluated with conventional indices of classification accuracy, and we focused on the proportion of abnormal superpixels and a surrogate measure of the false-positive rate. Last, we assessed the performance of SMAS in a sample with established glaucoma, while a key goal of en face imaging is early glaucoma detection, when conventional OCT metrics have been showed to be imperfect.^46^^–^^48^ As such, further evaluation in glaucoma groups with only the earliest signs of glaucoma would be useful. Nonetheless, we speculate that to detect the earliest changes, the examination of greater depths below the ILM would become even more pertinent than in our sample as more subtle defects may be more likely to be found deeper in the RNFL, possibly making SMAS more advantageous over its alternatives in earlier cases of glaucoma as supported by Figure 8.

In conclusion, we developed and presented a novel method for the construction and objective analysis of OCT en face slab images. The method considers all depths and regions containing visible RNFBs in healthy eyes, with the exception of the temporal raphe, as well as the individual anatomy of the eye. With this method, we are able to automatically and objectively detect glaucomatous changes of RNFB reflectance. In our glaucoma sample, this method outperformed other available approaches in detecting defects. Further assessment of this technique is warranted.

## Supplementary Material

Supplement 1

Supplement 2
