# Supplementary material for: Enhanced Objective Detection of Retinal Nerve Fiber Bundle Defects in Glaucoma With a Novel Method for En Face OCT Slab Image Construction and Analysis
Source: Transl Vis Sci Technol. 2021 Oct 4;10(12):1. doi: 10.1167/tvst.10.12.1 (PMC8496419; doi:10.1167/tvst.10.12.1)

Supplementary Figure 1

Different threshold levels (standard deviations below the grand mean intensity of visible RNFBs) evaluated to censor retinal regions with no visible RNFBs. 2.5 standard deviations below the grand mean intensity was selected as the final level. These regions can be seen in the corresponding panel as shades of grey, whereas regions in full black were ignored. SD = Standard Deviation.

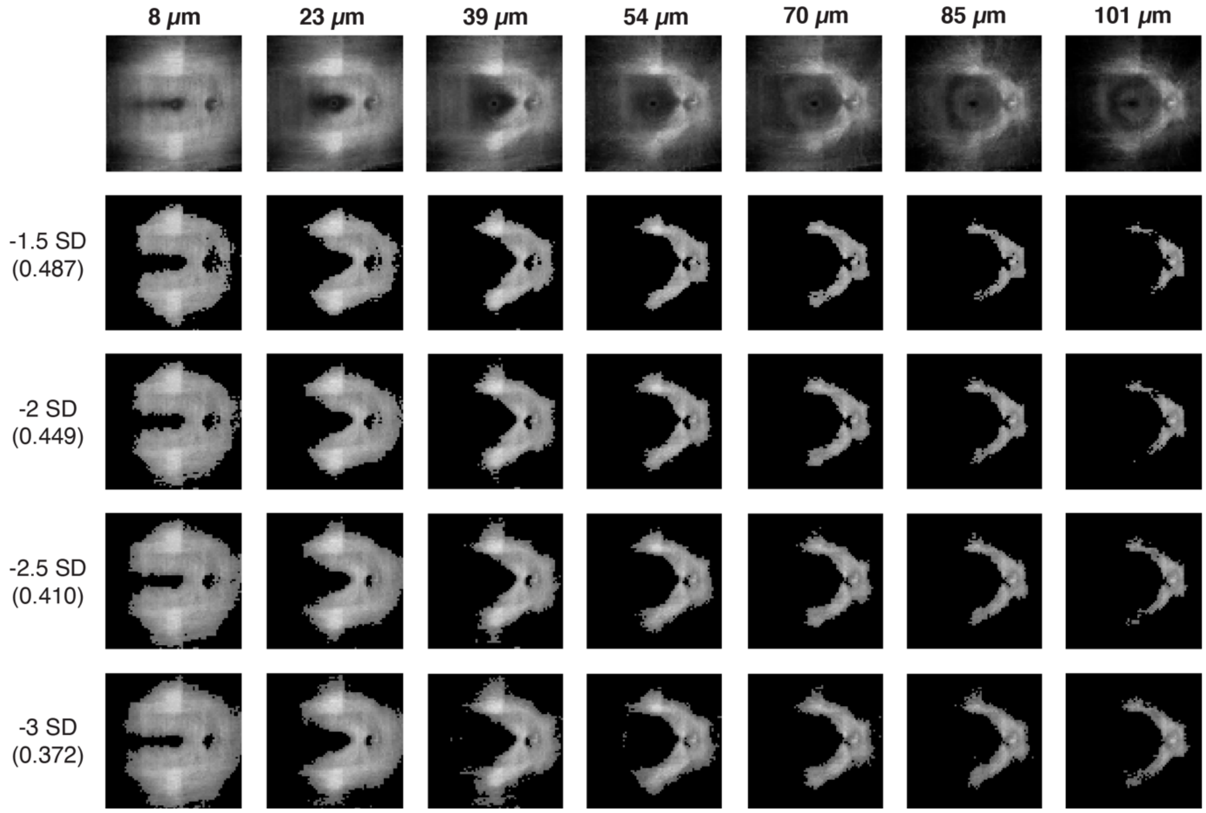

Supplement: Supplement 1 [file tvst-10-12-1_s001.pdf]
