# Supplementary material for: Enhanced Objective Detection of Retinal Nerve Fiber Bundle Defects in Glaucoma With a Novel Method for En Face OCT Slab Image Construction and Analysis
Source: Transl Vis Sci Technol. 2021 Oct 4;10(12):1. doi: 10.1167/tvst.10.12.1 (PMC8496419; doi:10.1167/tvst.10.12.1)
Supplement: Supplement 2 [file tvst-10-12-1_s002.pdf]

Supplementary Table 1

Detailed parameters of the 12 compounded slabs generated with the SMAS method.

**Slab n°**	From (µm below ILM)	To (µm below ILM)	Included in final analysis	**Slab n°**	From (µm below ILM)	To (µm below ILM)	Included in final analysis
**1**	7.8	23.2	Yes	**7**	100.7	116.1	Yes
**2**	23.3	38.7	Yes	**8**	116.2	131.6	No
**3**	38.8	54.2	Yes	**9**	131.7	147.1	No
**4**	54.3	69.7	Yes	**10**	147.2	162.5	No
**5**	69.8	85.1	Yes	**11**	162.6	178	No
**6**	85.2	100.6	Yes	**12**	178.1	193.5	No
